# Assessment of the interns’ ability based on Dundee model in Shiraz University of Medical Sciences

**Published:** 2015-10

**Authors:** MITRA AMINI, SAMANEH ABIRI, PARISA NABEIEI, SHIRIN GHANAVATI, ALI ASGHAR HAYAT, JAVAD KOJURI

**Affiliations:** 1 Education Development Center, Quality Improvement in Clinical Education Research Center, Shiraz University of Medical Sciences, Shiraz, Iran;; 2 Shiraz University of Medical Sciences, Shiraz, Iran

**Keywords:** Student, Assessment, Clinical Skills

## Abstract

**Introduction:**

The importance of medical profession and the role of the physician in society is no secret to anyone. Skills and competencies in clinical practice are necessary for the medical profession. In fact, in patient care, doctors require practical skills in addition to scientific knowledge. This study examines the potentials of medical school students in three areas of doing the right thing, doing the right thing in an intermediate range, and doing the right thing by the right person.

**Methods:**

This study was done in a descriptive-analytical and sectional model. The population of this study was all interns of Shiraz University of Medical Sciences who were passing internship at Internal Medicine, Surgery, Pediatrics, Obstetrics and Emergency wards. About 100 persons were selected by simple randomization. In order to collect data, a questionnaire with 12 questions was designed in two parts. The questionnaire was approved by 7 Faculty members of Clinical Medicine and Medical Education, and its reliability was approved by test-retest method on 20 medical students in the form of a pilot study and through Cronbach's alpha (82%). Collected data were analyzed by SPSS software version 14 using descriptive statistical methods.

**Results:**

Results showed that within the inner circle, interns evaluated their skills in surgery, internal medicine, and gynecology wards, intermediate and at other wards as weak. Also within the center circle, interns evaluated adequate educational evidence-based training in the field of medicine, and sufficiency of educational training in the field of clinical decision making and clinical care as suitable.

**Conclusion:**

According to the results, it seems that medical interns' skills in performing most medical skills are moderate. So teaching students by new educational methods and workshop techniques, using experienced teachers will be effective. The use of clinical skills training centers and objective assessment methods for the students' skills, especially before entering the clinical departments, is very important.

## Introduction

The value of medical profession and the effective role of the physician in society are no secret. Among the useful sciences, medicine has a special status and high dignity. This credit has emerged from this point that human life depends on maintaining and restoring his health (1). Medicine is not only a science, but also an art. Physician in patient care, in addition to scientific knowledge and human understanding, also requires practical skills. As physicians, should acquire the ability to apply theoretical knowledge and practical skills timely; they should also be able to establish clinical skills in dealing with patients and have appropriate communication and theoretical skills with their colleagues and patients. Thus clinical training in medicine is important and requires planning to empower the interns in order to conduct their duties as a physician (2).

Recent developments in the curriculum of general practitioners have a long history in the world. Academic training of general practitioners began since the 18th century. According to the study of the curriculum history of general practitioners, it is divided into five time periods. Training model, discipline-based model, organizations-based model (1765-1870), the model based on competencies (1971-1999), and evaluation-based model which continued till 1999. Modern medical education in Iran started since the establishment of the academy (Darolfonoon) in 1328 and for more than half a century it is still run based on discipline-based model and the traditional model which are not a good approach to deal with the challenges of the health system. Hence the need for an educational model which is consistent with the current situation in the society is greater than ever (3).

This educational model is the one that prepares physicians to carry out their duties in a society that is increasingly sophisticated. In this regard, it should be noted that over time, patients and the medical community have changed their expectations (4). In addition, training in clinical settings has been mixed with patients and their problems. In fact, the clinical environment is composed of admitted patients, ambulatory patients, and community. Therefore, teaching students to deal with real patients in clinical settings along with the problems of the day is necessary more than ever. Sometimes in clinical environment, different problems occur during routine care of patients.  John Spencer assessed common problems in the area of education in clinical settings, including lack of sufficient time for discussion and reflection of issues by the student-teacher, the presence of different levels of students in clinical settings (students’ internship), inappropriate physical environment for clinical education by the professors, patients’ issues, including short-term hospitalization, the patient's unwillingness to participate in the training program, etc., the absence of an appropriate system to monitor students and educational feedback (5).

According to the clinical environment and the need to use new methods in education, in this section, we explain the new educational model for training and evaluation of medical students. 

In 1999, at University of Dundee, Dundee Circular Model was designed for classifying learning outcomes and simpler assessment of required physician skills. It consists of three circles:

- Inner circle: do the right thing
- Center circle: do the thing by a right person
Circle of speech including social skills without which the physicians will not be able to carry out their duties. This includes understanding of social, psychological and medical aspects (6).
- The outer circle: Do the right thing by the right person. It includes one aspect of the medical profession which is dedicated to personal medical progress in his professional life. It has a control on central and internal circles.

The results of a study conducted by the World Health Organization were expressed as a "six feedbacks expected from physicians" in 2002. A person would be chosen as a doctor if he/she has acquired these skills: 1) Providing health care to the community, 2) Communication skills, 3) Treatment, 4) The power to make decisions in difficult conditions, 5) A suitable leader for health and treatment groups, and 6) Problem solving (7).

In a study on 60 faculty members from Tehran University, participants' views about the areas of physician competence which a graduate must achieve after studying course were collected and summarized. Capabilities in this area included clinical skills, communication skills, patient care (diagnosis, treatment, rehabilitation), health promotion and prevention, personal growth, professional and moral commitment and medical rights, decision-making skills, clinical reasoning and problem solving, health system, and the role of physicians (8).

Fuller and Smith designed a program to evaluate the tasks and skills of interns in the Brown Medical Sciences University in 1994; they divided the duties of the physicians into 9 categories: 1. Clinical skills like history taking, 2. Use of basic sciences knowledge in practice, 3. Diagnosis, prevention and treatment, 4. Effective communication, 5. Continuing education, 6. Personal growth, 7. Clinical ethics 8. Considering the community and its problems in addition to the clinical environment of hospitals and clinics, and 9. Problem solving ability.

In another study conducted at the University of Ontario, the researchers concluded that in the present society clinical education is not confined to the hospital environment and it includes the community and ambulatory patient clinics. In this study, it was shown that a part of the interns’ acquisition of skills is obtained through observation of faculty and residents' work. After that the student must obtain appropriate theoretical knowledge, behavioral skills and clinical judgment to participate actively in the diagnosis and treating patients. In this way, teachers and residents also play a role in addition to monitoring; during this learning process, interns learn professional values and responsibility in front of the patient, the society in which they live, and their colleagues (9).

In another study performed on "practical training for interns in Medical Sciences of Tabriz University," 20 basic techniques of available clinical training opportunities to perform any of the clinical procedures were examined. In this study, 200 interns were asked to complete the questionnaire. This study was performed using factor analysis; rotation and practical skills of interns were divided into three groups. In basic skills, the level of the interns’ ability was 51.4%. Most interns evaluated their skills poor to perform tracheal intubation, splinting, chest tube placement and removal of foreign bodies. Most procedures have been done through observation and supervised by professors (10).

## Methods

This is a descriptive, analytic and cross-sectional study. The study population included all interns of Shiraz University of Medical Sciences from 2004 to 2006 who were working in the areas of internal medicine, surgery, pediatrics, obstetrics and emergency of Nemazi, Shahid Faghihi, Hafiz and Dastgheyb hospitals. Among them, 100 interns were selected by simple randomization as the statistical sample (with the suggestion of a statistical consultant), using a list of medical students (that Assistance of Medical School researcher obtained) and the list of random numbers. The tool used for data collection was a questionnaire prepared by the researcher in two parts based on the Dundee model. The first part consisted of demographic questions and the second part contained 12 questions about the responsibilities of clinical students in three areas of doing the right thing, – doing the thing by the right person and doing the right thing by the right person. The first part of the questionnaire contained demographic questions (age, sex and year of entry to university) and the second part examined clinical skills in three circles of DUNDEE Model as below:

- 7 questions in the inner circle which assessed the students’ abilities to perform clinical procedure, clinical examination, history taking in surgical, women, internal and emergency wards.
- 2 questions in the middle circle which asked the students’ opinions about the adequacy of educational training in the field of clinical decision making, patient care and evidence-based medicine.
- 3 questions in outer circle which asked the students' opinions about medical ethics, responsibility of students about the patient and other partners as well as respecting the integrity in physical examination.

The validity of this questionnaire was reviewed and approved by 7 teachers experienced in Clinical Education and the Medical Education, in terms of wording, writing, repeated frills and appropriateness of questions. In order to evaluate the reliability of this questionnaire, the test-retest method was performed as a pilot study in 20 medical interns (as suggested by a statistical consultant's opinion) using Cronbach's alpha coefficient. Finally, α=82% was calculated. 

After the data were collected by the researcher, they were analyzed using SPSS software version 14 and descriptive and inferential statistical method. 

## Results

Based on the descriptive indicators, the subjects had a mean age of 24.61±0.8. Respondents included 54 males (54%) and 46 female (46%). 53% of the respondents were indigenous and 47% non-indigenous.

In response to the first research question, "How much are the Shiraz University of Medical Sciences’ students able to carry out the procedures of different wards (surgery, internal medicine, obstetrics and other wards)". As it can be seen in Table 1, most of the students had acquired the ability of suturing and wound closure procedure (64% of very good items) in the surgical ward. As to the procedures of tube insertion within the chest, 45% of students responded very weak; they had the lowest capacity in this sector.

As mentioned, in the case of gastric tube insertion procedure and washing it, they had the highest capacity (82% of them responded very well); it seems to be the lowest efficiency, among the procedures of this section.

In the obstetric ward, Pap smears had the best performance among the procedures in the women's section from students’ point of view (33% chose very good responses). 23% of the students responded very weak about episiotomy procedure, which had the lowest performance in the women's section procedure.

Also in the other wards, the students had the highest capacity in venous blood sampling (64% responded very well). But in the case of spinal fluid sampling only 1% of the students had responded well; this had the least performance in this ward. 

In response to the second research question, "What are the interns' capabilities in history taking and clinical examination?"; the results of the following table provide a review of this question.

In terms of skills in history taking and physical examination, internal ward had the best performance (49% responded "well"). In the surgical ward, 37% reported good status and 31% responded medium; it was the lowest skills of students in this ward.

In response to the third research question, "What are the interns' capabilities in the area of circles (inner, middle, outer)?"; the results of the following table provide a review of this question.

54% of interns evaluated evidence-based educational training in the medical fields as appropriate. 34% considered this training insufficient. Also, 52% of the students evaluated this training in clinical decision making and health care as appropriate.  And 33% considered it as inadequate. Also, 57% of the students believed that they always followed medical ethics. In the accountability and the scientific promotion, respectively 12% and 22% of the items were answered "always ". As shown in Table 1, the highest frequency in the field of interns' skill in surgery ward’s procedure with 33% belonged to medium, which represents the average performance of students from their own perspective. The average performance of students is 3.5 and the standard deviation is 1.2. In the field of skills in internal medicine ward, the students evaluated their effectiveness as medium (36% responded medium). The average study population was 4 and the standard deviation was 1.42. In the obstetrics ward, 29% of the students evaluated their effectiveness as medium, which reveals medium performance of the interns in this section. About the skills that students should be able to do in all wards, most students had poor efficiency. (31% selected the item “weak”.) In the field of history taking, students had the best performance in internal areas. 71% of the interns assessed their potential very well. In using new technology to improve their academic skills, 49% agreed. As shown in the Table below, 54% of the interns evaluated medical education in evidence-based training as appropriate. 34% considered this training as insufficient. 52% of the students selected “appropriate: for training in clinical decision making and health care and 33% regarded it as inadequate. 57% of the students believed that they always adhered to medical ethics.

As to the accountability and the scientific promotion, 50% and 39% respectively had responded very much, which has the highest frequency in the two areas (Table 1).

**Table 1:** Frequency distribution and percentage of respondents in three circles of Dundee model

**Answers**		**Disagree**	**No opinion**	**Agree**	**Total**	**Mean±SD**
		N (%)	N (%)	N (%)	N (%)	
**Inner circle**	Surgery	36 (36%)	31 (31%)	33 (33%)	100 (100%)	3.5±1.2
	Internal medicine	23 (23%)	43 (43%)	34 (34%)	100 (100%)	4±1.4
	Women	28 (28%)	44 (44%)	28 (28%)	100 (100%)	3.6±1.5
	Others	39 (39%)	25 (25%)	36 (36%)	100 (100%)	3.9±1.3
**Communication skill**	History taking and physical examination	2 (2%)	1 (1%)	97 (97%)	100 (100%)	4.6±0.5
	Communication with patients	11 (11%)	39 (39%)	50 (50%)	100 (100%)	3.5±1.03
	Use of technology	14 (14%)	27 (27%)	59 (59%)	100 (100%)	3.5±0.88
**Middle circle**	Educational teaching sufficiency based on EBM	34 (34%)	54 (54%)	12 (12%)	100 (100%)	1.8±0.67
	Educational teaching sufficiency in decision making and improving patients’ care	33 (33%)	52 (52%)	15 (15%)	100 (100%)	1.7±0.64
**Outer circle**	Medical ethics	7 (7%)	21 (21%)	72 (72%)	100 (100%)	4±1.03
	Accountability and personal-scientific development	31 (31%)	8 (8%)	61 (61%)	100 (100%)	3±1.3
	Responsibility	6 (6%)	32 (32%)	62 (62%)	100 (100%)	3.6±0.8

As shown in Table 2, the coefficient of determination of dependent variable in the internal area and related independent variables was 0.111, in the central area 0.08 and in the outer area 0.07. This indicates that the available effective variable largely failed to predict the dependent variable. It seems that there is a relationship between the ability of the students in performing tasks in Dundee model (Table 2).

**Table 2:** Regression Analysis of Independent Variable and Students’ Clinical Responsibilities of Dundee Model

	Multiple correlation coefficient	Coefficient of determination	Analysis of variance	p
**Inner circle**	0.33	0.11	1.97	0.09
**Middle circle**	0.27	0.08	1.3	0.25
**Outer circle**	0.27	0.07	1.33	0.25

**Table 3:** Correlation between independent variables and respondents’ clinical responsibilities in Dundee model

	**Age**	**Pre-intership score**	**Sex**	**Marriage status**	**Place of birth**
**Surgical procedures**	0.76	0.56	0.69	0.74	0.74
**Internal medicine procedures**	0.37	0.8	0.32	0.5	0.9
**Women procedures**	0.23	0.98	0.001	0.6	0.036
**Skill in other wards**	0.47	0.11	0.24	0.79	0.5
**History taking and physical examination**	0. 8	0.8	0.003	0.34	0.98
**Communication with patients**	0.27	0.26	0.16	0.4	0.8
**Use of technology**	0.2	0.93	0.7	0.7	0.5
**Educational teaching sufficiency based on EBM**	0.98	0.53	0.1	0.6	0.8
**Educational teaching sufficiency in decision making and improving patients’ care**	0.9	0.2	0.04	0.6	0.3
**Responsibility**	0.8	0.4	0.002	0.8	0.1
**Accountability and personal-scientific development**	0.5	0.2	0.4	0.8	0.8
**Medical ethics**	0.9	0.7	0.29	0.2	0.9

According to Table 3, there is a relationship between the age and the duties of interns in Dundee model; in the level of correlation it is low to moderate. Significance level of dependent variables with age was more than 0.05. Therefore, there was not a significant relationship between them. There was a relationship between the pre-internship and Interns’ duties in Dundee model that in the level of correlation it is weak. This relationship was at a significance level more than 0.05. So this relationship cannot be confirmed.

## Discussion

In the past, it was thought that physical education is unchangeable. But the three circles model of Dundee provided an opportunity in which necessary changes can be made in the clinical training system on the basis of social needs and cultural changes. Clinical training consists of four main components: knowledge, communication skills, problem solving skills, and physical examination (4).

According to a study conducted in 2002 by Sayari, clinical training in Iran has many defects and general practitioners have gained little skills in their clinical tasks (9-11). In this study, interns in practical skills at the internal area (doing the right thing) evaluated their average performance from weak to moderate. Such skills as blood sampling, stomach tube or urinary tube insertion were often rated acceptable by interns. These capabilities can be either performed by other hospital staff, especially nurses. On the other hand, in carrying out procedures that are vital and dealing with the patient life, the physician is allowed to do cardiac and pulmonary resuscitation, tracheal intubation, delivery, but in them they have very poor performance (12, 13).

In a study conducted at University of Sydney in Australia, it was observed that students who have had previous experiences in the field of skills performing, for the first time and under the supervision of assistant, had less anxiety and better performance. Also in another study in 2000, which was carried out by Arasteh, it was shown that there was a distance between the abilities that physicians have achieved and the desired level (11).

Evidence-based medicine means that physicians should learn the use of scientific evidence in various clinical aspects, especially in the field of evidence on the advantages and disadvantages of medical treatment. In the present study, it was found that physicians treat patients more from their experiences than using scientific and academic evidence. In this study, 54% of the students believed that education in the field of evidence-based medicine was sufficient. 34% believed that these trainings are inadequate. Unfortunately at the moment, despite the efforts made, even most of the physicians at the specialty level are not familiar with the concepts of evidence-based medicine (12, 13).

Studies showed that most of the interns' training occurs in hospital and this type of training does not make them familiar with the needs of the society. At hospitals, physicians are often encountered with patients who have chronic diseases and sometimes suffer from a rare disease. They are often trained in the specialized areas that include a small percentage of the patients in society, although knowledge of these diseases is essential, but not enough. In this regard, in ambulatory patients’ clinics, patients are more various. Thus interns are more familiar with the common diseases and health issues in the community. This is very important in terms of environment and conditions that will be encountered in the future and in terms of relation to other forces that are working in these centers (14).

In a study conducted in Isfahan, Interns had the highest level of satisfaction (39%) and in ambulatory patients’ clinics they had the lowest level of satisfaction (30%) of training and facilities in emergency departments. Interns show the highest rate of welcoming of ambulatory patients’ clinics. Now in a study conducted in Shiraz, 52% of the interns considered the ambulatory patients’ medical education as appropriate. 33% believed that the training rate in this section was inadequate. In this regard, more attention of authorities in education should be paid on filing and recording of patient information, education, disease prevention and coordination with other organizations involved in health (15).

For successful practice, knowing these skills is necessary, but not sufficient. Each physician should be familiar with the laws and regulations of his/her profession and should be sensitive to the professional ethics. Unfortunately, in practice, they do not pay much attention to moral education and medical secrets while all universities in today world teach it at specialty level. Interns learn communication with patients and fellows, medical ethics and accountability beside patients and by observing the behavior of assistants or professors (16). In this regard, 39% of the interns believed that they had not been able to communicate effectively with patients and patients' families. Also, 57% said they had always adhered to medical ethics in dealing with patients. It is important since enhancement of these skills will cause improvement in the students’ ability in history taking and physical examination and will increase the accuracy of diagnosis and patient confidence (17). Students need to save this issue in their mind from their first contact with the patient and try to fully implement and respect the patients’ rights; they also fully recognize their own rights and respect the profession by maintaining proper behavior and enhancing their knowledge (16, 18).

One of the other physicians’ tasks is trying to improve academic and individual promotion. In this study, the interns were satisfied to some extent about their performance. One way to increase the skills is to use technology such as the Internet and e-learning (19). Usually the cost of this type of education is low and based on previous studies the rate of learning in this way is the same. In this study, 49% of the students were satisfied about the educational aids for research and promotion of their academic level. However, in one study that was conducted in 2002, 83.3% of the respondents believed that they had little skill in the use of electronic resources and their facilities in this area had been limited. According to the research by Zahedi, the use of this resource will motivate the students to undertake research (11).

## Conclusion

According to the results, it seems that clinical skills of Interns are moderately proper in performing most of the medical procedures. So by educating students new and modern clinical education approaches, we can lead them towards effective care giving to patients and it can be also important to help the students’ acquire clinical skills before their entrance to major wards.

The results of this study indicated that there was a distance between the existing and proper situations which must exist in medical education. It seems that we need to empower the interns in order to fulfill their needs and enter society with high competency. In this way, the patients’ care can be improved through some measures such as establishing clinical skills centers and using active educational approaches should be considered. 
